# Incidence of total hip and total knee replacements from the prospective epidemiologic risk factor study: considerations for event driven clinical trial design

**DOI:** 10.1186/s12891-019-2680-3

**Published:** 2019-06-26

**Authors:** Cecilie L. Bager, Morten Karsdal, Asger Bihlet, Christian Thudium, Inger Byrjalsen, Anne C. Bay-Jensen

**Affiliations:** 1 ProScion, Herlev, Denmark; 2 Nordic Bioscience, Herlev, Denmark

**Keywords:** Total joint replacements, Osteoarthritis, Clinical trial design, Postmenopausal women, Register

## Abstract

**Background:**

Osteoarthritis (OA) leads to joint failure and total joint replacement (TJR, either hip (H) or knee (K)). Worsening of pain and joint space narrowing are believed to be surrogates for joint failure; however, we hypothesize that TJR, as a reflection of joint failure, can be used as an endpoint in event-driven clinical trials within a reasonable duration. We explored the incidence of TJR in the Prospective Epidemiologic Risk Factor (PERF I) study.

**Methods:**

A total of 5855 Danish postmenopausal women aged 49–88 enrolled in the PERF I study during 1999–2001 (baseline). Three-, six- and twelve-year follow-up data from the Danish National Patient Registry was collected, including occurrence of TJR and OA diagnosis. At baseline the women were asked whether they had OA.

**Results:**

The women with a TJR diagnosis before or after baseline were on average 1 year older (*p* < 0.001) and heavier (p < 0.001), compared to women with no TJR. The 3-, 6- and 12-year cumulative incidences were 1.1, 2.4 and 6.0% for TKR, and 2.1, 4.4 and 9.3% for THR. For those with an OA diagnosis at baseline the respective incidences were 2.7, 5.6 and 11.7% and 3.9, 7.2 and 13.6%

**Conclusions:**

Within 3, 6 or 12 years TJR incidences were double for women with an OA diagnosis compared to the *all-comer* population. TJRs are frequent amongst elderly women with OA and it is, therefore, feasible to conduct event-driven clinical trials where TJR is the endpoint demonstrating clinical benefit of a novel disease-modifying OA drug (DMOAD).

## Introduction

Osteoarthritis (OA) is the most common form of arthritis, affecting about 10% of the world’s population [1]; however without effective disease-modifying OA drugs (DMOADs) [2]. It is well accepted that the lack of DMOADs is partly due to the heterogeneity of OA, associated with a lack of effective enrichment strategies for clinical trial design [3, 4]. OA is considered a serious disease by the FDA [3, 5, 6] as OA is associated with higher mortality, which opens the venue for accelerated clinical trial designs, such as approval under subpart H. This poses new challenges and opportunities to drug developers, in terms of surrogate endpoints and reliable outcome measures proving clinical benefit in post approval studies.

The trajectory of OA progression has been described as interchanging stages of inertia and progression, emphasizing lack of linearity and large variation in clinical parameters [7]. Consequently, OA progression can span several decades, meanwhile have periods of rapid progression ultimately leading to joint failure and total joint replacement (TJR). The lack of clear clinical phenotypes and subgrouping tools results in large drug trials with expectations of marginal significant effect size. Traditionally, X-ray (e.g. joint space narrowing (JSN)) and psychometric tools (e.g. WOMAC) have been used as co-primary endpoints in pivotal drug testing trials: X-ray is an insensitive measure which requires 2 to 3 years to reach statistically significant differences between treatment groups [8], whereas psychometric readouts often are compromised by large placebo effect that mask treatment effects. While MRI may be a more sensitive measure to change than X-ray [9], their predictive value for TJRs are very similar [10]. FDA has recently issued a guideline regarding development of DMOADS, where they suggest that joint failure or TJR is the endpoint which the drug should protect against to be DMOAD [11]. Thus, there is a need to further understand and investigate the incidence of total hip and knee replacements in OAin populations to extend on other investigations relating risk factors for Incidence and prevalence of total joint [12], including the OMERACT initiative [13–15].

Accelerated approvals open the possibility for conducting outcome studies of which TJRs may be a preferred option. TJR is a validated clinical endpoint based on the Flemming criteria [6], while image scores such as JSN or cartilage volume have the possibility to be surrogate endpoints of TJR, although not validated. According to the aforementioned guideline from FDA, lack of progression assessed by an imaging biomarker has to translate directly to a clinical benefit for the patients to act as a surrogate endpoint [11]. A phase III clinical trial with an efficacious drug may be needed to establish and validate this connection. This underlines the need for understanding the prevalence and incidence of TJR to allow further trial design optimization.

The aim was to investigate the prevalence and incidence of total joint replacement in a high-risk population. Between 1999 and 2001, 5855 postmenopausal Danish women ages 48 to 89 years enrolled in the Prospective Epidemiologic Risk Factor (PERF I) study.

## Methods

### Study design

The Prospective Epidemiologic Risk Factor (PERF I) study is previously described by Neergaard et al. [16]. A total of 5855 Danish postmenopausal women included in the PERF I study during 1999–2001 (baseline). Women who had previously either participated in or were screened to participate in prevention trials at the Center for Clinical and Basic Research in Denmark were invited to participate in PERF I. The PERF I study was carried out in accordance with applicable regulatory and ethical guidelines, and the study protocol was approved by the local ethics committees. All participants signed an informed consent.

### Baseline investigations

At baseline, the participants completed an interview with a doctor or a nurse. The questions included: smoking status (daily smokers (never/previous/current)), alcohol consumption (≥7 drinks/week (yes/no)), walking (≥10 min/week (yes/no)), level of education (elementary/high school/ university), OA diagnosis (yes/no), rheumatoid diagnosis (RA) (yes/no), cardiovascular diseases (CVD) (yes/no) and treatment of osteoporosis (OP) (yes/no). Vital signs were also collected at baseline.

### Description of cases

In Denmark, every citizen has a unique personal identification number (CPR number) that enables matching of individuals to health registries. Diagnosis of OA and incidence of TJRs were extracted from the Danish National Patient Registry and the Danish National Diabetes Register at 31-12-2014 (end of study). Diagnosis were classified according to WHOs International Classification of Diseases 8 (ICD8) from 1977 to 1993 and WHOs International Classification of Diseases 10 (ICD10) from 1994. TJR were classified according to the Old Danish Operations and Treatment Classification for Operations from 1977 to 1995 and the Danish version of NOMESCO Classification of Surgical Procedures for 1996. Date of death and date of emigration were extracted from the Danish Death Registry and CPR Registry at end of study.

In this paper, the following diagnosis and surgeries were defined as a positive diagnosis in the interview or the following registry-codes: osteoarthritis (OA): ICD10: DM15–19, ICD8: 713, RA: ICD10: DM05, DM06, DM08; ICD8: 712, OP; ICD10: DM80-DM82; ICD8: 7230, diabetes mellitus: inclusion in the Danish Diabetes Register, CVD: ICD10: chapter 10, ICD8: 390–450, total knee replacements (TKR): ICD10: KNGB, ICD8: 7004 and 8278 and total hip replacements (THR): ICD10: KNFB, ICD8: 7003 and 8274. In this manuscript only primary THR or TKR were included in the manuscript.

### Statistics

Baseline characteristics of women with and without a THR or TKR were compared using a student t-test for numerical variables and a chi-square test for categorical variables.

The age-specific incidence rate of THR and TKR was calculated for each 5-year group as the average annual number of new cases per 10.000 women alive at the start of each age group. Age-specific prevalence of THR and TKR was calculated as the average annual number of new and preexisting cases per 10.000 women alive at the start of that age group.

Time period incidence rate of THR and TKR from baseline until 3, 6 and 12 years after baseline was calculated for the entire cohort and women with OA at baseline. It was calculated dividing the total number of THR and TKR during the different time periods by the population at baseline.

Cumulative incidence of new THR and TKR after baseline in the total cohort and in women with OA at baseline was calculated using Kaplan-Meier estimates.

The statistical analyses were performed using, R software v. 3.4.1 (R Development Core Team, 2012) and GraphPad Prism v. 7.01 (GraphPad Software, La Jolla, USA)

## Results

A total of 5855 postmenopausal women were included in the PERF I study that were previously described by Neergaard et al. [16]. Of the women enrolled in PERF I, 430 women had a total of 566 TKR, 786 women had a total of 985 THR and 1106 women had either a TKR or a THR until end of study. Furthermore, a total of 133 women had a THR at baseline and 42 women had a TKR at baseline. Table 1 summarizes the baseline characteristics of the entire cohort stratified by women with (cases) or without a TKR or a THR (non-cases).

**Table 1:** Patient Characteristics at baseline

Cohort Characteristics	Women with TJR* Cases *n* = 1106		Women without a TJR* Non-cases *n* = 4749		
	%	OA	%	No.	*p*-value
Age at baseline, years					
Mean (No.)	71.2 (n = 1106)		70.1 (n = 4749)		< 0.001
SD	6.39		6.57		
BMI					
Mean (No.)	27.4 (*n* = 1066)		25.9 (*n* = 4571)		< 0.001
SD	4.45		4.17		
Daily Smoking (yes/no)					0.001
Never	49.8	550/1105	46.8	2217/4740	
Current	18.4	202/1105	23.5	1113/4740	
Previous	31.9	353/1105	29.7	1410/4740	
Alcohol (≥7 drinks/week)	31.9	350/1097	32.7	1546/4724	0.61
Walking (≥10 min/week)	90.5	1000/1105	91.6	43,742/4739	0.25
Education:					0.65
Primary school	72.1	797/1105	71.4	3381/4736	
High school	21.4	237/1105	21.34	1013/4736	
University	6.4	71/1105	7.2	342/4736	
Osteoarthritis	59.8	661/1106	28.1	1332/4749	< 0.001
Rheumatoid Arthritis	3.8	42/1106	3.3	155/4749	0.62
Osteoporosis	9.7	107/1106	12.1	577/4749	0.02
Diabetes mellitus	4.9	54/1106	3.7	175/4749	0.08
sCardiovascular disease	50.8	565/1106	48.0	2279/4749	0.08

Abbreviations: BMI, Body Mass Index; min, minutes;

*Women diagnosed with a TKR or THR before 2015

### Incidence and prevalence of total joint replacement

**Fig. 1:**
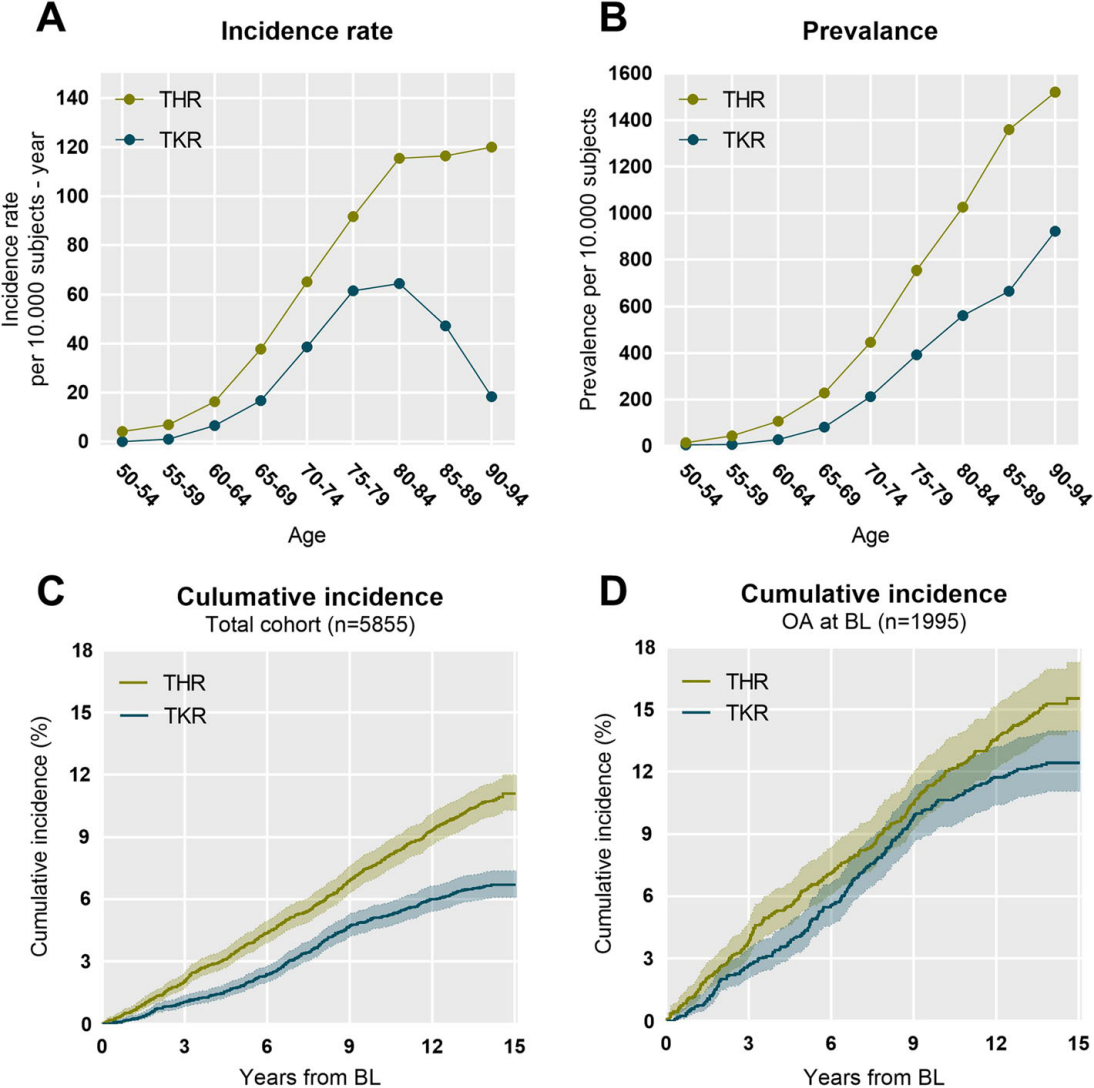
**a)** Age specific incidence rate of total hip replacement (THR) and total knee replacements (TKR). **b**) Age specific prevalence of THR and TKR. **c**) Kaplan-Meier estimates of the cumulative incidence and 95% CI of THR and TKR in the total cohort **d**) Kaplan-Meier estimates of the cumulative incidence and 95% CI of THR and TKR in with OA at Baseline

The prevalence increased with age and more steeply for THR than for TKR. The prevalence of TKR increased from the age of 50–54 until the age of 90–94 where the average yearly prevalence reached 920 per 10.000 population (Fig. 1b). The prevalence of women living with a THR increased from the age of 50–54 until the age of 90–94 where the prevalence reached 1520 per 10.000 population. The yearly average incidence rate increased with age until age 80–84 for TKR where the incidence rate reached 64 per 10.000 population and started to decline. The yearly average incidence rate increased for THR until the age of 80–84 where the incidence rate plateaued at 115 per 10.000 population (Fig. 1a).

Next, we investigated the cumulative incidence of new THR and TKR after baseline in two different subgroups of women 1) the all-comer population and 2) women with an OA diagnosis at baseline. The Kaplan-Meier estimate of the cumulative incidence of TKR among the total population after 3, 6 and 12 years was 1.1, 2.4, and 6.0%, respectively. The cumulative incidence of THR was estimated to be 2.1, 4.4, and 9.3% after 3, 6 and 12 years, respectively (Fig. 1c). For the OA population, the cumulative incidence of TKR was estimated to be 2.7, 5.6, and 11.7% and the cumulative incidence of THR was estimated to be 3.9, 7.2 and 13.6% 3, 6 and 12 years after baseline, respectively (Fig. 1d).

## Discussion

Clinical trial design for the development of treatments for OA is constantly evolving, in response to new biological discoveries and the regulatory environment. There is a need to understand the incidence of TJR in different sub-populations to evaluate the feasibility of event-driven clinical trials, as part of the accelerated approval process by FDA under subpart H.

We found that the incidence of THR and TKR increased from 1 to 2% after 3 years and from to 6–9% after 12 years, indicating that this population consisting of elderly women has a significant risk of developing joint failure and undergoing TJR. TJR was associated with BMI, Age, smoking status, osteoporosis as well as borderline significantly with of cardiovascular and diabetes, which is in alignment with other studies [12, 17].

Not surprisingly, the incidence was markedly higher in the women with an OA diagnosis at baseline (2–4% after 3 years). A 2–4% incidence rate over a 3-year period may seem low for clinical studies based on event rates. However, in osteoporosis studies, yearly incidence rates between 3 and 5% are within the event range for events [18] requiring a sample size of typically more than a total of 1650–4000 patients treated for approximately 1.5–3 years to reach statistically significant treatment effects between two treatment groups, depending on the expected effect size of the intervention [19]. Assuming a linear annual TKR incidence rate of 0.5% in the reference group over a seven-year study duration, and an expected risk reduction of 50% of the intervention, a sample size of approximately 1800 participants per treatment group would be needed to detect a significant difference in a two-sided test with an alpha of 0.05 and a power of 90%.

In the Osteoarthritis Initiative (OAI), the 2- and 4-year cumulative incidences of TKR were found to be 5.1 and 11.7%, respectively in a population consisting mainly of OA patients with a Kellgren-Lawrence grade of at least 2 or prior TJR [20]. This is markedly higher than what we observed in our all-comer population, however, expected as only a targeted patient population with definite radiographic OA was included from the OAI cohort. Other important studies have investigated the risk factors associated with of total hip and knee replacements in OA, in which similar risk factors were identified as compared to the current study [12, 17]. To assist in clinical practise and studies, guidance for TJRs in have been developed by the OMERACT initiative [13–15]. Interestingly the OARSI-OMERACT Task Force on total joint replacement, reported that: “Although symptom levels were higher in patients recommended for TJR, pain and functional disability alone did not discriminate between those who were and were not considered to need TJR by the orthopaedic surgeon” [14]. This highlights the need to identify risk factors in subpopulations with OA at higher risk for TKR to enrolled in clinical studies, and the need for the development of objective guidance criteria for such impart patient affecting decisions. Surgery should be reserved for those that have not responded appropriately to less invasive methods [21], as TKRs are associated with revisions [22] and complications [23].

At present more than 670,000 total knee replacements are performed annually in the US [24]. There is a significant proportion of patients whom experience the need for revision surgery. Albeit several modifiable risk factors have been identified with revisions of TKR [25], more than twice as many patients have pain after revision surgery compared with patients after primary TKA [26], and patients after revision TKA surgery have reduced function, quality of life as compared with patients after primary TKA surgery [26]. These combined notions further emphasises and warrants further research to understand the biological and psychological criteria of TJRs, both in clinical practice and for the optimal design of clinical studies. The OMERACT initiative have achieved a patient supported guidance [27–30] for TJRs, which may assist in the harmonization and understanding of TJRs in clinical practice and clinical trials.

In OA, total joint replacement (TJR) is considered joint failure. As a comparator, in osteoporosis, another musculoskeletal disease, event-driven studies are required for standard approval [18]. Patients are included based on e.g. prevalent osteoporotic fractures and/or low bone mineral density (a surrogate measure) and other risk factors, while the approval is based on a relative event reduction. As a commonly used tool to estimate the risk of vertebral fractures in osteoporosis trials, the FRAX© tool is often used in determining a suitable sample size [31]. The FRAX© algorithm is based on a selection of clinical risk factors and bone mineral density at the femoral neck. In terms of event-driven outcomes in OA, recent study of Tanezumab, indicated a higher incident rate of TJRs in the tanezumab treated group with concomitant NSAID [32, 33], suggesting that TJR as an outcome may be meaningful in clinical research settings. With the current and emerging knowledge of risk factors for structural progression and joint replacement in OA, it may be possible to develop a similar tool for use in OA trials to aid in the determination of a suitable sample size for the given study population. While these data suggest that TJR may be used as an outcome measure in clinical trials, it is well-known that a TJR is not only based on objective decision-making, but also subjective individual evaluation in which factors with substantial inter-person variation affect the decision as well as being confounded by the doctor-patient interaction. Consequently, there is a need to standardize the TJR decision-making process and provide guidelines that may be used in clinical settings.

## Conclusion

In this study we found that within 3, 6 or 12 years TJR incidences were double for women with an OA diagnosis compared to the *all-comer* population. TJRs are therefore frequent amongst elderly women with OA and the data support the possibility of using TJR as a hard endpoint in clinical studies to demonstrate the clinical and patient-centric clinical benefit.

Authors’ contributions.

Conception and study design: CLB, ACBJ, MK, CT and AB. Data collection: MK and IB. Data analysis and interpretation: CLB, MK, AB, ACBJ. Drafting of article or revising content: CLB, MK, AB, ACBJ, IB, CT. Final approval of the article: CLB, MK, AB, ACBJ, IB, CT.

## Data Availability

The dataset generated and/or analyzed during the current study are not publicly available do to legal and ethical reasons but are available from the corresponding author on reasonable request.

## Abbreviations

CVD: Cardiovascular diseases
DMOAD: Disease-modifying OA drug
ICD10: International Classification of Diseases 10
ICD8: International Classification of Diseases 8
JSN: Joint space narrowing
OA: Osteoarthritis
OAI: Osteoarthritis Initiative
OP: Steoporosis
PERF: Prospective Epidemiologic Risk Factor
RA: Rheumatoid diagnosis
THR: Total hip replacements
TJR: Total joint replacement
TKR: Total knee replacements
